# Acceptance and Commitment Therapy modulates immuneinflammatory responses and neurotrophic factors homeostasis in elderly stroke patients: A randomized controlled trial

**DOI:** 10.5937/jomb0-58244

**Published:** 2025-11-05

**Authors:** Jing Wang, Haiyan Gu, Xiaorong Hu, Yanjie Zhou, Lingling Wu

**Affiliations:** 1 Ningbo Rehabilitation Hospital, Center for Cognitive Psychological Rehabilitation, Ningbo, Zhejiang, 315000, China; 2 Ningbo Rehabilitation Hospital, Department of Intensive Care Rehabilitation, Ningbo, Zhejiang, 315000, China; 3 Ningbo Rehabilitation Hospital, Department of Scientific Research, Ningbo, Zhejiang, 315000, China; 4 Ningbo Rehabilitation Hospital, Department of Pain Medicine, Ningbo, Zhejiang, 315000, China; 5 Ningbo Rehabilitation Hospital, Department of Neurorehabilitation, Ningbo, Zhejiang, 315000, China

**Keywords:** cerebral stroke, T lymphocyte subsets, inflammatory, neurotrophic factors, oxidative stress, moždani udar, podskupine T limfocita, inflamatorni, neurotrofni faktori, oksidativni stres

## Abstract

**Background:**

This study examined the regulatory effects of Acceptance and Commitment Therapy (ACT) on T lymphocyte subsets, serum inflammatory cytokines, neurotrophic factors, antioxidant enzymes, and lipid peroxidation products in elderly cerebral stroke (CS) patients, providing insights into the multi-dimensional pathophysiological interactions and potential intervention strategies for chronic stroke recovery.

**Methods:**

In this randomized controlled trial, 120 elderly stroke patients were allocated to either an ACT group (ACT intervention; n = 60) or a routine group (conventional treatment; n = 60). Comprehensive assessments were performed to quantify: (1) peripheral T lymphocyte distribution (CD3+, CD4+, CD8+ subsets, and CD4+/CD8+ ratio), (2) serum inflammatory cytokines (IL-1p, IL-6, IL-10, and TNF-a), (3) neurotrophic factors (5-HT, NE, BDNF, and IGF-1), and (4) antioxidant enzymes (SOD, CAT) and lipid peroxidation products (MDA, NO) using flow cytometry, HPLC-ECD, and ELISA. Statistical analyses were conducted with SPSS 22.0.

**Results:**

Following treatment, CS patients exhibited reduced CD3+ and CD4+ T-cell levels along with a decreased CD4+/CD8+ ratio, while CD8+ T-cell proportions were elevated (P&lt; 0.05). Proinflammatory cytokine levels (IL-1 b, IL-6, and TNF-a) were significantly suppressed, whereas anti-inflammatory IL-10 expression increased (P &lt; 0 .0 5 ). Notably, ACT demonstrated superior efficacy in restoring immune balance and attenuating inflammation compared to conventional intervention (P&lt; 0.05). Furthermore, neurotrophic factors levels were elevated, and oxidative stress markers were ameliorated in CS after treatment (P&lt; 0.05), suggesting that ACT enhances neurotrophic activity and mitigates oxidative injury.

**Conclusions:**

ACT likely confers neuroprotection through multi-target mechanisms, including modulation of T-cell subset homeostasis, upregulation of neurotrophic factors, and suppression of oxidative stress.

## Abstract (sr)

**Uvod:**

Ova studija je ispitala regulatorne efekte terapije prihvatanja i predanosti (ACT) na podskupine T limfocita, serumske inflamatorne citokine, neurotrofične faktore, antioksidativne enzime i proizvode peroksidacije lipida kod starijih pacijenata sa cerebralnim moždanim udarom (CS), pružajući uvid u multi-dimenzionalne strateške potencijalne interakcije. oporavak od moždanog udara.

**Metode:**

U ovom randomizovanom kontrolisanom ispitivanju, 120 starijih pacijenata sa moždanim udarom raspoređeno je ili u ACT grupu (ACT intervencija; n = 60) ili u rutinsku grupu (konvencionalni tretman; n = 60). Izvršene su sveobuhvatne procene da bi se kvantifikovalo: (1) periferna distribucija T limfocita (CD3+, CD4+, CD8+ podgrupe i CD4+/CD8+ odnos), (2) serumski inflamatorni citokini (IL-1 p, IL-6, IL-10 i TNF-a), (3) neurotropni faktori (5-HT, NE, BDNF i IGF-1), i (4) antioksidativni enzimi (SOD, CAT) i proizvodi peroksidacije lipida (MDA, NO) korišćenjem protočne citometrije, HPLC-ECD i ELISA. Statističke analize su sprovedene sa SPSS 22.0.

**Rezultati:**

Nakon lečenja, pacijenti sa CS su pokazali smanjene nivoe CD3+ i CD4+ T-ćelija zajedno sa smanjenim odnosom CD4+/CD8+, dok su proporcije CD8+ T-ćelija bile povišene (P&lt; 0,05). Nivoi proinflamatornih citokina (IL-1 b, IL-6 i TNF-a) su značajno potisnuti, dok je ekspresija antiinflamatornog IL-10 povećana (P&lt; 0,05). Posebno, ACT je pokazao superiornu efikasnost u obnavljanju imunološke ravnoteže i ublažavanju upale u poređenju sa konvencionalnom intervencijom (P&lt; 0,05). Takođe, nivoi neurotrofičnih faktora su bili povišeni, a markeri oksidativnog stresa su poboljšani u CS nakon tretmana (P&lt; 0,05), što sugeriše da ACT pojačava neurotrofičnu aktivnost i ublažava oksidativnu povredu.

**Zaključak:**

ACT verovatno daje neuroprotekciju kroz više ciljne mehanizme, uključujući modulaciju homeostaze podskupa T-ćelija, povećanje neurotrofnih faktora i supresiju oksidativnog stresa.

## Introduction

The global aging population has intensified the public health burden of cerebral stroke (CS), particularly among individuals aged 65 and older, who account for over 75% of cases [1]. Epidemiological studies reveal alarming disability and mortality rates of 50% and 30%, respectively, imposing substantial socioeconomic and healthcare challenges [2]. While therapeutic advances such as thrombolysis and endovascular interventions have improved outcomes, elderly patients continue to face poor functional recovery due to multimorbidity, immune senescence, and diminished neuroplasticity [3]. Consequently, elucidating novel pathophysiological mechanisms and developing targeted rehabilitation strategies for geriatric CS have emerged as critical research priorities.

Mounting evidence underscores the pivotal role of immune-inflammation in CS pathogenesis [4]. Dysregulation of T-cell subsets (e.g., CD4, CD8 , regulatory T cells [Tregs]) exacerbates secondary neuronal injury and impairs recovery in CS by disrupting immune homeostasis [5]. Concurrently, fluctuations in inflammatory cytokines (e.g., IL-6, TNF-α, CRP) correlate with post-stroke neuroinflammatory severity and patient outcomes [6]. Furthermore, disruptions in neurotrophic factors (e.g., 5-HT, DA, GABA) not only contribute to post-stroke depression and cognitive impairment but may also influence recovery by modulating neuroimmune interactions [7]. However, current research remains largely confined to acute-phase biomarker analyses [8] [9], with limited systematic investigation into the dynamic immune-neurotrophic factors interplay during chronic rehabilitation or the regulatory effects of rehabilitative interventions—a knowledge gap impeding precision therapy development. Acceptance and Commitment Therapy (ACT), an emerging cognitive-behavioral intervention, enhances psychological flexibility to promote adaptive behaviors and emotional regulation in patients with chronic conditions, yet its application in elderly CS rehabilitation remains exploratory [10]. Notably, psychological interventions may modulate immune-inflammatory responses and neurotrophic factors release via hypothalamic-pituitary-adrenal axis regulation and autonomic nervous system modulation, thereby facilitating post-stroke neural repair [11]. Nevertheless, empirical evidence characterizing ACT's impact on the immune-neuroendocrine axis in geriatric CS remains absent.

This pioneering investigation represents the first systematic effort to integrate ACT intervention with dynamic monitoring of immunoneuroendocrine biomarkers (including T lymphocyte subpopulations, serum inflammatory cytokines, and neurotrophic factors profiles), aiming to elucidate the potential biological pathways of psychosocial interventions and provide a theoretical foundation for a multidimensional rehabilitation model for elderly CS. By bridging the gap between psychological interventions and biomarker research, this work transcends the traditional biomedical paradigm. These findings provide a scientific foundation for integrating ACT into a psychobiological framework for elderly CS patients, holding significant clinical translational value.

## Materials and methods

### Study population

**Figure 1:**
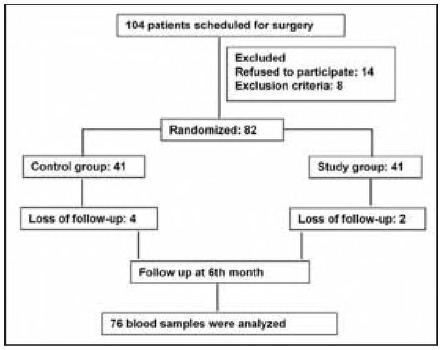
Flow Chart.

We enrolled 120 elderly CS patients who received rehabilitation therapy in our hospital's Neurology and Rehabilitation Department between September 2022 and April 2024. All participants were diagnosed via cranial magnetic resonance imaging (MRI) or computed tomography (CT). Inclusion Criteria: (1) Meeting established diagnostic criteria for geriatric stroke with disease duration of 2 weeks to 1 month; (2) Age 60 years at enrollment; (3) Presence of residual limb motor dysfunction; (4) Preserved consciousness and cognitive function, without communication barriers; (5) Willingness to participate in this research with signed informed consent from both patients and their families. Exclusion Criteria: (1) Concurrent Alzheimer's disease; (2) Pre-existing neuropsychiatric conditions (e.g., depression, anxiety, personality disorders) or family history of mental illness; (3) Severe comorbidities (e.g., cardiopulmonary failure, malignant tumors). Using a random number table, patients were assigned to either the ACT group (n = 60) treated with ACT or the regular group (n = 60) given conventional therapy. Randomization was performed using stratified block randomization (block size=4), with allocation concealment achieved via sealed opaque envelopes, all patients and data collectors were unaware of their subgroups. There were 39 males and 21 females in the ACT group with age (68.57±5.36) years; 42 males and 18 females in the regular group with age (68.07±4.23) years. Figure 1 shows the detailed process of collection and grouping of study subjects. The study protocol was approved by Ningbo Rehabilitation Hospital ethics committee (Approval number: 2022-16-G1). All participants provided informed consent while remaining blinded to group allocation.

### ACT

The ACT protocol comprised 12 weekly sessions (60 min each), including mindfulness exercises, value clarification, and committed action planning. Sessions were led by certified ACT therapists, with adherence monitored via session recordings and therapist logs.

### Sample collection and processing

Fasting venous blood samples were obtained at baseline and post-treatment. Each sample was aliquoted into three portions for analysis of T lymphocyte subsets, serum inflammatory cytokines, antioxidant enzymes, lipid peroxidation products, and neurotrophic factors.

### Laboratory tests

Peripheral venous blood (2 mL) was collected from participants using EDTA anticoagulant tubes and maintained at room temperature for processing within 6 hours. Flow cytometry analysis was performed as follows: 100 pL of whole blood was incubated with fluorescently conjugated monoclonal antibodies [fluorescent antibodies included CD3-FITC (clone SK7), CD4-PE (clone RPA-T4), and CD8-APC (clone SK1) (BD Biosciences)] for 15 minutes in the dark. Erythrocytes were lysed using a lysis buffer, followed by centrifugation and resuspension in PBS. Samples were analyzed on a BD FACSCanto II flow cytometer (flow cytometer calibration was performed daily using BD Calibrite beads, with compensation adjustments via single-stained controls), and lymphocyte subsets were quantified using FlowJo v10 software. The percentages of CD3^+^ (total T lymphocytes), CD4^+^ (helper T cells), and CD8^+^ (cytotoxic T cells) were determined, along with the CD4^+^/CD8^+^ ratio. Absolute counts of lymphocyte subsets were determined using Trucount tubes (BD Biosciences), following manufacturer protocols.

An additional 3 mL of venous blood was collected and allowed to clot for 30 minutes at room temperature. Serum was separated by centrifugation (3000 rpm, 15 min, 4°C), aliquoted, and stored at -80°C until analysis. Serum neurotrophic factors were quantified using high-performance liquid chromatography with electrochemical detection (HPLC-ECD). Briefly, samples were filtered through 0.22 μm membranes and separated on a C18 reverse-phase column with a mobile phase (95: 5 phosphate buffer [pH 3.0] : methanol) at 1.0 mL/min flow rate. Analyte quantification of 5-HT and NE by electrochemical detector.

Serum concentrations of interleukin (IL)-1β, IL-6, IL-10, tumor necrosis factor-α (TNF-α), brain-derived neurotrophic factor (BDNF), insulin-like growth factor-1 (IGF-1), superoxide dismutase (SOD), malondialdehyde (MDA), catalase (CAT), and nitric oxide (NO) were measured using enzyme linked immunosorbent assay (ELISA) kits (IL-βb : LK-E0012, IL-6: LK-E0023, IL-10: LK-E0031, TNF-α: LK-E0045; Hangzhou Lianke Biotechnology Co., Ltd., batch No. 20230521) according to the manufacturer's protocols.

### Questionnaires

Patients were administered the Acceptance and Action Questionnaire-II (AAQ-II) [12], Cognitive Fusion Questionnaire (CFQ) [13], Hospital Anxiety and Depression Scale (HADS-A/HADS-B) [14], and Edmonton Symptom Depression Questionnaire (ESDQ) [15] questionnaires, with lower scores indicating better psychological status of patients.

### Statistical analysis

Statistical analyses were conducted using SPSS22.0. Continuous variables (e.g., age, CD3^+^, and CD4^+^ cell counts) were first assessed for normal distribution using the Shapiro-Wilk normality test. Within-group comparisons (pre- *vs.* post-treatment) were analyzed using paired t-tests, while between-group differences were assessed via independent samples t-tests. Mixed-effects models were applied for longitudinal data analysis. All statistical tests were two-tailed, with p-values <0.05 considered statistically significant.

## Results

### Alterations in T lymphocyte subsets

At baseline, there was no statistically significant difference in the detection of T lymphocyte subsets between the two groups (P>0.05). Compared with baseline, patients' CD3^+^, CD4^+^, CD4^+^/CD8^+^ were higher after treatment, while CD8^+^ was lower (P<0.05). Among them, CD3^+^, CD4^+^, CD4^+^/CD8^+^ were higher in ACT group after treatment than regular group, while CD8^+^ was lower than regular group (P<0.05, Table 1).

**Table 1:** Changes in T lymphocyte subsets. Note: Compared with baseline data, *P<0.05.

Groups (n = 60)		Conventional	ACT	t	P
CD3^+^ (%)	Baseline	25.71±4.50	25.96±4.06	0.312	0.756
	After	28.71±4.11*	32.11±6.75*	3.338	0.001
CD4^+^ (%)	Baseline	41.15±5.44	41.77±7.15	0.532	0.596
	After	45.37±6.16*	48.15±5.50*	2.611	0.010
CD8^+^ (%)	Baseline	29.38±4.38	29.78±5.10	0.451	0.653
	After	25.91±4.83*	23.78±4.28*	2.557	0.012
CD4^+^/CD8^+^	Baseline	1.43±0.28	1.46±0.43	0.420	0.675
	After	1.81±0.41*	2.09±0.45*	3.595	<0.001

### Changes in serum inflammatory cytokine profiles

In terms of inflammatory factors, it was seen that the levels of pro-inflammatory factors IL-1β, IL-6 and TNF-α were lower than those at baseline in both groups after treatment, while the level of anti-inflammatory factor IL-10 was higher (P<0.05). Comparison between groups showed that IL-1β, IL-6 and TNF-α were lower in the ACT group than in the REGULAR group after treatment, while IL-10 was higher than in the REGULAR group (P<0.05, Table 2).

**Table 2:** Changes in serum inflammatory factors. Note: Compared with baseline data, *P<0.05.

Groups (n = 60)		Conventional	ACT	t	P
IL-1β (pg/mL)	Baseline	33.65±4.45	33.66±5.22	0.008	0.993
	After	26.07±4.95*	23.12±5.36*	3.126	0.002
IL-6 (pg/mL)	Baseline	41.34±3.26	40.75±4.88	0.779	0.437
	After	30.10±4.23*	28.36±4.62*	2.150	0.034
IL-10 (pg/mL)	Baseline	2.05±0.79	2.11±0.59	0.463	0.644
	After	3.16±0.65*	3.44±0.59*	2.464	0.015
TNF-α (pg/mL)	Baseline	56.06±6.32	55.44±6.50	0.529	0.598
	After	37.52±6.38*	34.35±5.29*	2.959	0.004

### Fluctuations in neurotrophic factors levels

There was no difference in the comparison of neurotrophic factors test results at BASELINE between the two groups (P>0.05). After treatment, NE, 5-HT and BDNF were elevated in both groups; among them, the ACT group was higher than the regular group (P<0.05). In contrast, IGF-1 was lower in both groups after treatment than at baseline, with the ACT group being lower than the regular group (P<0.05, Table 3).

**Table 3:** Changes in neurotrophic factors. Note: Compared with baseline data, *P<0.05.

Groups (n = 60)		Conventional	ACT	t	P
NE (ng/mL)	Baseline	27.82±4.25	28.26±5.32	0.507	0.613
	After	42.42±4.55*	45.01±5.71*	2.754	0.007
5-HT (ng/mL)	Baseline	171.38±34.09	176.94±35.90	0.870	0.386
	After	267.48±39.83*	283.92±39.90*	2.259	0.026
BDNF (ng/mL)	Baseline	8.55±1.15	8.57±0.91	0.104	0.917
	After	12.13±3.49*	13.40±2.80*	2.196	0.030
IGF-1 (ng/mL)	Baseline	44.08±4.89	45.76±7.42	1.463	0.146
	After	35.94±4.79*	33.56±5.56*	2.515	0.013

### Variations in antioxidant enzymes and lipid peroxidation products

Detecting the oxidative stress of the patients, it was seen that NO and MDA were lower and SOD and CAT were elevated after treatment compared to at baseline (P<0.05). Compared to regular group, NO and MDA were lower and SOC and CAT were higher in ACT group after treatment (P<0.05, Table 4).

**Table 4:** Changes in oxidative stress response. Note: Compared with baseline data, *P<0.05.

Groups (n = 60)		Conventional	ACT	t	P
NO (μmol/L)	Baseline	34.56±4.36	34.60±4.60	0.0.49	0.961
	After	27.22±3.22*	25.34±4.84*	2.485	0.014
MDA (mmol/L)	Baseline	15.22±2.29	15.63±2.52	0.927	0.356
	After	9.41±2.96*	8.20±3.30*	2.107	0.037
SOD (U/L)	Baseline	82.08±8.27	83.92±8.09	1.226	0.223
	After	98.13±11.26*	104.62±12.73*	2.956	0.004
CAT (U/L)	Baseline	56.63±6.14	55.23±6.87	1.184	0.239
	After	63.08±5.85*	66.31±8.15*	2.492	0.014

### Changes in questionnaires

Finally, the changes in the results of the patients' questionnaires were compared, and it was seen that the AAQ-II, CFQ, HADS-A, HADS-B, and ESDQ scores of the two groups were lower than those at baseline after the treatment, of which the ACT group was even lower than that of the REGULAR group (P<0.05, Table 5).

**Table 5:** Changes in questionnaires. Note: Compared with baseline data, *P<0.05.

Groups (n = 60)		Conventional	ACT	t	P
AAQ-II	Baseline	26.28±6.26	26.48±6.09	0.177	0.860
	After	22.28±5.29*	20.42±4.48*	2.085	0.039
CFQ	Baseline	26.23±4.32	26.88±4.00	0.855	0.394
	After	22.85±4.30*	20.28±4.17*	3.317	0.001
HADS-A	Baseline	8.88±3.93	9.32±3.29	0.655	0.514
	After	6.95±2.50*	6.12±1.85*	2.075	0.040
HADS-B	Baseline	9.60±3.27	9.40±2.81	0.359	0.720
	After	6.93±2.05*	5.92±1.53*	3.078	0.003
ESDQ	Baseline	13.08±3.41	13.42±2.63	0.600	0.550
	After	10.28±2.43*	9.37±2.02*	2.249	0.026

## Discussion

The pathophysiology of senile CS encompasses a complex interplay of immune-inflammatory dysregulation, neuroendocrine disturbances, and oxidative stress damage. Through comprehensive analysis of dynamic alterations in T lymphocyte subpopulations, serum inflammatory cytokines, neurotrophic factors profiles, and oxidative stress indicators, this study elucidates the multifaceted biological dysregulation characteristic of elderly CS patients and investigates potential therapeutic targets of ACT.

In this study, it was observed that CD3^+^, CD4^+^, CD4^+^/CD8^+^ were elevated while CD8^+^ was decreased in patients after treatment, and T-lymphocyte subsets showed dynamic changes. These dynamic shifts in T lymphocyte subsets were accompanied by attenuated systemic inflammation, implying coordinated pathogenic interactions between T cell polarization and inflammatory cascade activation. Notably, the diminished CD3^+^ T cell count, representing total T cell depletion, may indicate systemic immunosuppression, whereas hyperactivated CD4^+^ T cell subsets appear to function as pivotal mediators of sustained pro-inflammatory responses [16]. Dysregulation of T lymphocyte subsets triggers IFN-γ secretion, activating macrophages and microglia and driving the production of IL-1β and TNF-α. These proinflammatory cytokines further compromise the blood-brain barrier (BBB), promoting neutrophil infiltration into the brain parenchyma and exacerbating cerebral edema and neuronal apoptosis [17]. Specifically, CD3^+^ and CD4^+^ T cells stimulate endothelial cells to upregulate adhesion molecules (e.g., ICAM-1) via IL-17, facilitating leukocyte extravasation and reactive oxygen species (ROS) generation [18]. In contrast, CD8^+^ T cells contribute to neuronal damage through distinct mechanisms: during the acute phase, they induce cytotoxicity in the ischemic penumbra via the perforin/granzyme pathway, while in the chronic phase, they may promote delayed neuronal death through Fas/FasL-mediated apoptosis [19]. Of particular significance, Cui H et al. demonstrated that Treg (CD4^+^, CD25^+^, FoxP3^+^) depletion leads to diminished IL-10 secretion, impairing NF- B pathway suppression and perpetuating a self-amplifying inflammatory cascade [20]. This Th1/Th17/Treg imbalance, characterized by elevated IL-6 and TNF-α alongside reduced IL-10, establishes a detrimental »inflammation-immunosuppression« cycle, which is a key determinant of poor clinical outcomes in elderly CS patients.

Second, in the present study, the observed elevation in neurotrophic factors levels coupled with a reduction in antioxidant enzymes and lipid peroxidation products following treatment in CS patients indicates a potential interplay between neurotrophic factors-neurotrophic axis dysregulation and oxidative stress pathways. The post-CS neurotrophic factors system imbalance not only impedes neurological rehabilitation but also establishes a reciprocal modulation network with oxidative stress processes [7]. Specifically, the depletion of 5-HTergic neurons results in the disruption of raphe nucleus-prefrontal cortex projection, which triggers depression-like phenotypes and attenuates hippocampal neurogenesis through downregulation of BDNF expression [21]. Concurrently, diminished NE levels compromise the locus coeruleus-noradrenergic system's regulatory control over microglial activity, thereby enhancing pro-inflammatory cytokine (IL-6 and TNF-α) secretion while suppressing antioxidant enzyme (e.g., SOD) production [22]. The reduction in key neurotrophic factors (BDNF and IGF-1) exerts direct inhibitory effects on both PI3K/Akt and MAPK/ERK signaling cascades, ultimately impairing synaptic plasticity and hindering neovascularization [23]. The observed reduction in IGF-1 post-treatment may reflect ACT-mediated suppression of stress-induced IGF-1 hypersecretion, as chronic stress is known to dysregulate IGF-1 signaling. However, further studies are needed to clarify its role in neural repair. Meanwhile, altered antioxidant enzyme sand lipid peroxidation products (diminished SOD and CAT activities and elevated MDA and NO levels) contribute to neural deterioration via diverse pathological mechanisms. For example, the reduction in MDA, a lipid peroxidation end-product, reflects attenuated oxidative damage to mitochondrial membranes. This may improve mitochondrial dynamics (e.g., fusion/fission balance) by preserving membrane integrity, thereby enhancing ATP synthesis and neuronal survival [24]. Reduced CAT activity leads to hydrogen peroxide accumulation, activating the JNK/p38 MAPK pathway and inducing neuronal apoptosis [25].

Finally, through the questionnaire survey, we observed that the psychological status of patients in both groups was effectively improved. Combined with the results above, we believe that ACT may improve biological disorders from the following pathways: (1) Immunomodulatory pathway: ACT reduces sympathetic nerve tone by alleviating psychological stress. (2) Neurotrophic factors-neurotrophic axis remodeling: ACT upregulates prefrontal cortex 5-HT and NE levels, which in turn activates BDNF-TrkB signaling and promotes the expression of synaptic proteins (e.g. PSD-95). In addition, ACT may enhance vascular endothelial growth factor (VEGF) expression through the IGF-1R/PI3K pathway and promote vascular neovascularization in ischemic areas [26]. (3) Oxidative stress antagonistic effect: ACT may reduce glucocorticoid-induced mitochondrial splitter protein expression and improve mitochondrial dynamics by inhibiting HPA axis overactivation [27]. Meanwhile, psychological intervention upregulates the Nrf2 signaling pathway, promotes the synthesis of antioxidant enzymes such as HO-1 and SOD, and inhibits NOX4-mediated ROS generation [28]. The decrease of MDA and the recovery of SOD activity in the ACT group in this study may be related to this mechanism. Consistent with Martinez-Calderon et al. [11], our findings demonstrate ACT's efficacy in reducing proinflammatory cytokines. However, unlike prior studies focused on chronic pain, this trial uniquely integrates immune and neurotrophic biomarkers, advancing ACT's application in stroke rehabilitation. Meanwhile, our findings are consistent with the AHA/ASA guidelines emphasizing multimodal rehabilitation [29]. In the future, ACT may synergize with exercise therapy by reducing inflammation-induced neurotoxicity, thereby facilitating motor recovery.

Although this study has preliminarily uncovered disruptions in the immune-neuro-oxidative stress network among elderly CS patients, several limitations should be acknowledged: (1) The relatively small sample size (n = 120) may limit statistical power to detect subtle effects (e.g., subgroup differences) and increase the risk of type II errors. At the same time, the short duration of follow-up resulted in our inability to assess the long-term prognosis of the patients. (2) Biomarker assessments were restricted to specific post-intervention time points, precluding a comprehensive evaluation of dynamic changes across the acute, subacute, and chronic phases of CS. (3) The mechanistic insights into ACT were derived primarily from peripheral blood biomarkers, with no direct examination of brain-specific alterations—such as localized immune cell infiltration, neurotrophic factors release, or mitochondrial function (e.g., via cerebrospinal fluid analysis or animal models). Consequently, the precise biological targets of ACT remain to be elucidated. (4) Although antiplatelet agents (e.g., aspirin) were administered uniformly across groups, their potential immunomodulatory effects could not be fully controlled. Future studies should stratify analyses by medication use. (5) Although Th17/Treg imbalance was hypothesized to underlie immune dysregulation, Th17-specific markers (e.g., IL-17, RORγt) were not measured, limiting mechanistic interpretation.

## Conclusion

In elderly post-CS patients, a self-reinforcing pathological network emerges, characterized by immune-inflammatory dysregulation, neurotrophic factors depletion, and oxidative stress, ultimately impairing neural recovery. ACT may improve the psychological status of CS patients by restoring T lymphocyte homeostasis, up-regulating BDNF/IGF-1 expression, attenuating oxidative damage.

## Dodatak

### Consent to publish

All authors gave final approval of the version to be published.

### Highlight

Acceptance and Commitment Therapy (ACT) significantly modulated T-cell subsets (↑CD3^+^/CD4^+^, ↓ CD8^+^) and rebalanced pro-/anti-inflammatory cytokines (↑IL-1β/IL-6/TNF-α, IL-10), highlighting its immunomodulatory potential in elderly stroke patients.

ACT upregulated key neurotrophic factors (5-HT, NE, BDNF) linked to synaptic plasticity and neural repair, while suppressing stress-related IGF-1 dysregulation, suggesting dual benefits in psychological and neurological recovery.

ACT reduced lipid peroxidation markers (MDA, NO) and enhanced antioxidant enzyme activity (SOD, CAT), demonstrating robust protection against oxidative damage in post-stroke neurodegeneration.

Improved psychological flexibility via ACT correlated with attenuated neuroinflammation and oxidative stress, bridging psychosocial interventions with measurable biomarker-driven outcomes for the first time.

This study pioneered the integration of ACT with dynamic monitoring of immune-inflammatory, neurotrophic, and oxidative stress biomarkers, offering a novel framework for precision rehabilitation in stroke care.

### Availability of data and materials

The data that support the findings of this study are available from the corresponding author upon reasonable request.

### Funding

This study was supported by the 2023 Zhejiang. Province Medical and Health Youth Innovation Project (No.2023RC086).

### Acknowledgements

Not applicable.

### Conflict of interest statement

All the authors declare that they have no conflict of interest in this work.
